# The increased prevalence of keloids in atopic dermatitis patients with allergic comorbidities: a nationwide retrospective cohort study

**DOI:** 10.1038/s41598-021-03164-4

**Published:** 2021-12-08

**Authors:** Hyo-Eun Kwon, Hye-Jin Ahn, Su Jin Jeong, Min Kyung Shin

**Affiliations:** 1 Department of Dermatology, College of Medicine, Kyung Hee University, 23, Kyungheedae-ro, Dongdaemun-gu, Seoul, 02447 Korea; 2 Medical Science Research Institute, Kyung Hee University Medical Center, Seoul, Korea

**Keywords:** Epidemiology, Immunological disorders

## Abstract

Atopic dermatitis (AD) is associated with allergic comorbidities, such as asthma, allergic rhinitis (AR), and allergic contact dermatitis (ACD). The etiology of keloid is largely unknown; however, AD and keloid share inflammatory pathways characterized by T-helper cell 2 cytokines and increased dermal fibroblast activity. The prevalence of keloids has been reported to increase in patients with AD, but it remains controversial. This study aimed to estimate the prevalence of keloids in patients with AD, and compare it with the prevalence of other comorbidities of AD. We assessed the Korean National Health Information Database and medical records including coexisting asthma, AR, and ACD. Single and multiple logistic regression models were created for keloids and each allergic disease. The prevalence of keloids was higher in the AD group than in the control group. Among patients with AD, adolescents and adults had a higher prevalence of keloids than infants and children. The risk of keloids was high with AD alone, and coexisting asthma significantly increased the risk. Similarly, the risk of keloids was higher in AR associated with AD and ACD associated with AD than in AD alone. Thus, among Koreans, patients with AD have a higher risk of keloid development, with coexisting allergic diseases increasing the risk.

## Introduction

Atopic dermatitis (AD) is a chronic inflammatory skin disease associated with numerous atopic and nonatopic comorbidities^1^. The prevalence of AD is estimated to be 15–20% in children and 1–3% in adults, and the incidence has increased two- to three-fold during the past few decades in industrialized countries^2^.

Keloid is a fibroproliferative dermal tumor resulting from skin trauma and subsequent abnormal scar formation^3^. The mechanism underlying the pathological development of keloids is largely unknown; however, it is mainly characterized by an overproduction of extracellular matrix and high activity of fibroblasts during the inflammatory and remodeling phases of scar formation, resulting in the excessive accumulation of collagen^4,5^. These processes are also observed in fibroblasts among patients with AD^6^. Although the association between AD and keloids is still unclear, several possible links have been hypothesized.

AD and keloid share common inflammatory pathways that are characterized by T-helper cell 2 (Th2) cytokines. An impaired inflammatory response of fibrogenesis involving Th2 cytokines secreted by CD4 + T cells such as interleukin (IL)-4, IL-5, IL-10, and IL-13, leads to the imbalance between pro-fibrotic and anti-fibrotic signals^7^. Additionally, in AD, upregulation of Th2 cytokines is triggered, and IL-4, IL-5, IL-10, and IL-13 are secreted, resulting in epidermal thickening, inflammation, pruritus, and decreased expression of barrier proteins such as filaggrin and loricrin, all of which are clinical characteristics of AD^8^. The reason why prurigo nodularis is frequently observed in patients with AD, with lichenification being one of the most common clinical manifestations of AD, is presumed to be due to this process^9^.

It is well established that patients with AD have a higher chance of developing allergic comorbidities such as asthma, allergic rhinitis (AR), and allergic contact dermatitis (ACD)^1^. Furthermore, the severity of AD was associated with a higher prevalence of comorbid allergic disorders and increased severity of comorbidities^10^. Based on these facts, the authors hypothesized that the patients with both AD and allergic comorbidities might have a higher prevalence of keloids.

Keloids occur more frequently in dark-skinned individuals, with a higher prevalence in Fitzpatrick skin phototypes III to VI^11^. African Americans have a 5 to 16 times higher prevalence of keloids than Caucasians^12^. The annual keloid incidence rate is 0.15% among the general Taiwanese population, but the exact prevalence among Asians is unknown^13^. In a large-scale retrospective cohort study in Taiwan, the probability of keloid development in patients with AD is 3.19 times higher than in the healthy population^14^. However, to date, there are no known reports on the prevalence of keloids in AD accompanied by allergic diseases.

To the best of our knowledge, this study first analyzed big data from the Korean National Health Information Database (NHID) and aimed to (1) estimate the prevalence of keloids in patients with AD and (2) compare the prevalence of keloids with those of patients with AD alone and those with both AD and allergic comorbidities.

## Materials and methods

### Data sources

The data used in this study were obtained from the Korean NHID, in which diseases were coded according to the International Classification of Diseases, Tenth Revision, Clinical Modification (ICD-10-CM). This database contains nationwide claims data and is linked at an individual level with the Korean registration number assigned to all Korean residents at birth or immigration for practical purposes. This study was approved by the Institutional Review Board of Kyung Hee University (IRB number 2019-07-076) and a waiver of informed consent was approved from IRB due to the retrospective nature of the big datasets. All methods were performed in accordance with the relevant guidelines and regulations.

### Study design and population

We assessed the Korean NHID from 2017 to 2018 and medical records were analyzed according to the ICD-10-CM codes. From this database, two groups were defined: an AD patient group and a control group. A total of 2,517,401 subjects under the ICD-10 code L20 were included in the AD group. For the control group, we excluded patients with the AD code, L20, for the past 3 years from the database. A random sample of 3% was prepared. In total, 3,132,283 subjects in the control group were matched by age and sex. All subjects in both groups were classified into two subgroups, with or without keloids. A diagnosis of keloids was defined as the condition under ICD-10 code L91.

Demographic information (age and sex) and comorbidities of allergic diseases identified by ICD-10-CM codes were also collected. The associated allergic diseases in this study included asthma (ICD-10 code J304), AR (ICD-10 code L209), and ACD (ICD-10 code L239). All patients and the control group were classified by age: infant and early childhood, 0–5 years; late childhood, 6–10 years; adolescence, 11–20 years; adulthood, 21–60 years; and the elderly, > 61 years.

### Statistical analysis

A chi-square test was employed, and the odds ratios (ORs) were analyzed to compare the AD and control groups by age and sex. In the age group, the OR of late childhood, adolescence, adulthood, elderly was compared with infancy and early childhood as a reference. Also, in the sex, the OR of female was compared as male as a reference. In addition, logistic regression was performed to analyze the differences in the effects of variables, including the presence or absence of AD, age, and sex, on the prevalence of keloids. Furthermore, we analyzed the OR of the risk of keloids between the AD patients without asthma (or allergic rhinitis, allergic contact dermatitis) and with asthma (or allergic rhinitis, allergic contact dermatitis), and was compared to the control group through the multiple logistic regression adjusted by age and gender. A *p*-value of < 0.05 was considered statistically significant. All data processing and statistical analyses were performed using SAS Enterprise Guide 7.1 (SAS Institute Inc., Cary, NC).

### Ethical approval

This article does not contain any studies with human participants or animals performed by any of the authors. This research study was conducted retrospectively from data obtained for clinical purposes. An IRB of Kyung hee university hospital officially granted ethical approval.

## Results

### Baseline characteristics of patients

Baseline characteristics of patients in the AD and control group are shown in Table 1. All personnel in this study were Koreans. A total of 5,649,684 personnel were included in this study, and 44.56% (2,517,401) had AD (see Supplementary Table S1 online). In patients with AD, 26.01% of patients were younger than 5 years, and 51.88% were younger than 20 years of age. The rate of AD tended to decrease with age. Of the patients with AD, 53.01% were females. Among all age groups, the prevalence of AD was the highest among infants and children.

**Table 1:** Baseline characteristics of patients with AD and control group.

Variables	Atopic dermatitis (n = 2,517,401)	Controls (n = 3,132,283)	OR	95% CI	*p*-value
**Age group, n (%)**					
Infancy and early childhood	654,796 (48.29)	701,299 (51.71)	1.000		
Late childhood	329,795 (45.34)	397,638 (54.66)	0.888	0.883–0.893	< 0.001
Adolescence	321,596 (43.62)	415,745 (56.38)	0.828	0.824–0.833	< 0.001
Adulthood	877,329 (42.70)	1,177,245 (57.30)	0.798	0.795–0.802	< 0.001
Elderly	333,885 (43.12)	440,356 (56.88)	0.812	0.808–0.817	< 0.001
**Sex, n (%)**					
Men	1,182,857 (44.80)	1,457,194 (55.20)	1.000		
Women	1,334,544 (44.34)	1,675,089 (55.66)	0.981	0.978–0.985	< 0.001

Baseline characteristics of patients in the AD and control group. All personnel in this study were Koreans.

The *p*-value was obtained through the chi-square test.

*CI* confidence interval.

### Increased prevalence of keloids in the AD group

The prevalence of keloids was higher in the AD group than in the control group (Table 2). Keloids exist in 1.43% (36,090 out of 2,517,401) of the patients with AD and in 0.52% (16,216 out of 3,132,283) of the control group. Simple logistic regression was performed, and the prevalence of keloids was significantly higher among the patients with AD than in the control group (OR 2.795, *p* < 0.001). When comparing the prevalence of keloids according to the age groups of all patients, the prevalence of keloids was higher among adolescents and adults than among those belonging to infancy and early childhood (OR 18.333, and OR 29.289, 95%, *p* < 0.001). The entire numbers can be found as Supplementary Table S2 online.

**Table 2:** Prevalence of keloids in patients with and without AD.

Variables	N	Keloid (%)	OR	95% CI	*p*-value
Control	3,132,283	16,216 (0.52)	1.000		
AD	2,517,401	36,090 (1.43)	2.795	2.743–2.847	< 0.001
**Age group, n (%)**					
Infancy and early childhood	1,537,360	4108 (0.27)	1.000		
Late childhood	857,092	6463 (0.75)	2.836	2.727–2.949	< 0.001
Adolescence	968,628	45,350 (4.68)	18.333	17.755–18.930	< 0.001
Adulthood	3,520,791	256,188 (7.28)	29.289	28.399–30.208	< 0.001
Elderly	1,343,210	37,627 (2.80)	10.757	10.415–11.110	< 0.001
**Sex, n (%)**					
Men	3,868,370	147,039 (3.80)	1.000		
Women	4,358,711	202,697 (4.65)	1.234	1.226–1.243	< 0.001

The effect of presence or absence of AD on the risk of keloids was evaluated using the simple logistic regression.

*AD* atopic dermatitis, *CI* confidence interval.

### The effect of allergic comorbidities associated with AD on keloids

The effect of having allergic comorbidities associated with AD on the risk of developing keloids (Table 3) was evaluated using multiple logistic regression. Patients with AD alone were at a higher risk of keloids (OR 2.97, *p* < 0.001), and asthma coexisting with AD increased the risk of keloids significantly (OR 3.17, *p* < 0.001). This effect was also observed in AR and ACD. In patients with AR associated with AD, the risk of keloids increased compared with that in AD alone (OR 3.2, vs. OR 2.61, *p* < 0.001). In patients with ACD associated with AD, the risk of keloids increased compared with that in AD alone (OR 3.81, vs. OR 2.32, *p* < 0.001). The entire numbers can be found as Supplementary Table S2 online.

**Table 3:** OR of keloids according to the type of allergic diseases associated with AD.

Variables	Classification	OR	95% CI		*p*-value
Asthma	Control	1.000			
	AD without asthma	2.968	2.911	3.026	< 0.001
	AD with asthma	3.171	3.080	3.264	< 0.001
AR	Control	1.000			
	AD without AR	2.606	2.542	2.673	< 0.001
	AD with AR	3.196	3.133	3.259	< 0.001
ACD	Control	1.000			
	AD without ACD	2.324	2.273	2.376	< 0.001
	AD with ACD	3.813	3.735	3.892	< 0.001

The effect of having allergic comorbidities associated with AD on the risk of keloids was evaluated using multiple logistic regression adjusted for age and sex.

*AD* atopic dermatitis, *AR* allergic rhinitis, *ACD* allergic contact dermatitis, *CI* confidence interval.

## Discussion

This is the first study demonstrating that the risk of keloids is strongly associated with AD in the Korean population. Our results also demonstrate that the prevalence of keloids increases with coexisting allergic diseases, such as asthma, AR, and ACD. Previous data on the prevalence of keloids is based mainly on Caucasians and Taiwanese in Asia^13,14^. In Korea, where all citizens are obligated to subscribe to national medical insurance, large cohort study using electronic medical information is feasible. For the first time, our findings show that the prevalence of keloids increased in patients with AD in the Korean population. Hajdarbegovic et al. also suggested that keloids may be strongly associated with atopic asthma in European, African, and Asian populations^15^. The findings in this study are consistent with those of previous studies wherein the risk of keloids is higher in AD patients with allergic comorbidities.

The molecular pathophysiology of keloid formation and progression is poorly understood. However, fibroblasts, one of the main cells responsible for most of the collagen and extracellular matrix deposition in both normal and abnormal wound healing, have been identified to contribute to keloid formation and dermal structural changes in AD skin lesions^16^. Recently, Shin et al. reported that the expression of thymic stromal lymphopoietin (TSLP), an IL-7-like cytokine, was increased in keloid tissue compared to the normal skin. In addition, keloidal fibroblasts treated with TSLP produced higher levels of collagen I, collagen III, and transforming growth factor β compared to normal fibroblasts, which explains the pivotal role of TSLP in keloid development^17^. Increased serum TSLP has also been observed in AD patients^18^.

Another molecule that contributes to the collagen production of dermal fibroblasts in AD is Oncostatin M (OSM), a T lymphocyte/macrophage-derived proinflammatory signaling molecule similar to the IL-6 cytokine family^19^. Fibroblasts are target cells for OSM. OSM stimulates collagen and glycosaminoglycan production in dermal fibroblasts, and this process can be observed in both keloidal formation and prurigo nodularis in patients with AD^20^.

Other than upregulation of Th2 cytokines, effector memory CD8 + T cells and CD103 + CD8 + resident memory T (TRM) cells are increased in keloid tissue. Therefore, increased CD8 + TRM in keloid tissues might contribute to local inflammation^21^. TRM cells have been shown to contribute to the recurrence of AD; however, the exact roles of skin TRM are still unclear^22^.

There have been a few reports on the association between allergic diseases and keloid development; however, these results are still debated*.* Hajdarbegovic et al., for example, revealed no association between AD and keloids in the adjusted model of a case–control study. Additionally, they suggested that asthma was significantly, consistently, and strongly associated with keloids. However, there were no consistent associations found on keloids with atopic eczema or hay fever^15^. In contrast, Lu et al. demonstrated that patients with AD had a greater possibility of developing keloids in a nationwide retrospective cohort study in Taiwan. According to this study, the AD cohort had significantly higher percentages of patients with asthma (25.41 vs. 12.91, *p* < 0.001), AR (54.78 vs. 35.92, *p* < 0.001), and allergic conjunctivitis (57.22 vs. 42.08, *p* < 0.001) compared with the non-AD cohort^14^. In our study, AD patients with ACD also had an increased prevalence of keloid risk, which is different from previous studies, suggesting that ACD may have the characteristic of AD-associated comorbidity. In the past, the higher permeation of contact allergens through the disrupted skin barrier was the main factor of ACD in patients with AD. Recently, potential shared immune pathways have been demonstrated for subsets of AD and ACD, including Th1, Th2, Th9, and/or Th17^23,24^.

Han et al. demonstrated the relationship between a disintegrin and metalloprotease 33 (ADAM33) polymorphism and keloid scars in an East Asian population. Q-1 SNPs in blood were significantly associated with keloid scars. ADAM33 protein is a zinc-dependent endopeptidase, characterized by a pro-domain, metalloprotease domain, and disintegrin domain^25^. The ADAM33 gene was found to be associated with asthma and airway hyperresponsiveness^26^. It is thought that this may explain the increased keloid risk in AD associated with asthma. Considering the literature mentioned above, AD and keloid share various genetic and pathologic mechanisms.

One of the strengths of this study is the use of a large population-based claims dataset consisting of one ethnic group, which enabled the analysis of all cases of AD, keloids, and comorbid allergic diseases. Moreover, since the Korean NHID is one of the largest claims datasets including all age groups and the entire region, the possibility of selection bias is reduced to compare the relationships between the AD patients and control group. Second, it is the first time that a large cohort analysis has demonstrated the result of an increase in the prevalence of keloids in AD patients with allergic diseases. However, this study has several limitations. First, due to the characteristics of the nationwide database of the NHID, the possibility of false negatives exists in both groups. All participants were patients who visited the hospital for any purpose. Therefore, it cannot be ruled out that both groups have a slightly higher likelihood of keloid diagnosis compared to the actual healthy group, which increases the possibility of underestimation compared to the actual prevalence of keloid. Second, there may be a relative underestimation of the prevalence of keloids in the control group, the general patient population. Compared to patients with AD who visit a dermatologist, the control patients who visit other departments are likely to be unaware of their keloid scars and have no need for medical services. However, considering the accessibility and affordability of medical specialists in the Republic of Korea, this statistical difference is thought to be small compared to other countries. Third, since the data are large-scale cohort data collected for insurance purposes rather than research purposes, several important confounding factors including family history, occupation and level of education were not provided. Thus, further prospective studies with accurate inclusion criteria are needed to clarify the exact relationship between allergic diseases and keloids.

In conclusion, this study demonstrated that in the Korean population, patients with AD have a higher risk of keloid development, and the risk is even higher with coexisting allergic diseases. Nevertheless, further exploration of this association is needed to confirm these relationships and the pathophysiological mechanisms common to AD and keloids.

## Supplementary Information

Supplementary Information.

## Data Availability

All databases are available as Supplementary Information files.
